# The relationship between submaximal measures of cardiorespiratory fitness, brain structure, and cognition in older adults

**DOI:** 10.1002/alz.093192

**Published:** 2025-01-09

**Authors:** Mickeal N Key, Sydney White, Rebekah Zweydoff, Tara Hawes, Sandra A Billinger, Amanda Szabo‐Reed, Eric D Vidoni

**Affiliations:** ^1^ University of Kansas Alzheimer’s Disease Research Center, Fairway, KS USA; ^2^ University of Kansas Medical Center, Kansas City, KS USA

## Abstract

**Background:**

Cardiorespiratory fitness (CRF) has been repeatedly linked to healthy brain and cognitive outcomes in aging. Treadmill‐based graded maximal exercise testing (GXT) is a common technique for measuring CRF in exercise studies. Because it is often challenging for older adults to reach the commonly accepted criterion measure of oxygen consumption, alternate, submaximal measures have been explored. The relationship between submaximal assessments of CRF, cognition, and brain health has not been extensively studied. One that tracks well with peak oxygen consumption (VO_2 peak_) is the oxygen uptake efficiency slope (OUES), the regression slope between maximum oxygen consumption and the log of minute ventilation. We hypothesize that OUES will be associated with 1) medial temporal, parietal, and occipital structural brain regions and 2) attentional control, processing speed, and visuospatial function.

**Method:**

In an ongoing exercise trial,163 older adults (age = 71.06 (4.18), 73.1% female, 19.2% from historically under‐represented populations) underwent baseline testing. As part of a broader physical function assessment, peak VO_2peak_ was measured during a treadmill‐based GXT, and OUES was calculated based on 100% of exercise time. Structural MRI scans and scores from a comprehensive neuropsychological battery were also collected.

**Result:**

VO_2peak_ and OUES were positively associated with several hippocampal, medial temporal, and occipital volumetric measures, with VO_2peak_ only tracking with gray matter segments and OUES tracking with both gray and white matter segments of these regions. Overall, the associations with brain volume were stronger for OUES than for VO_2peak_. Both VO_2peak_ and OUES were positively associated with visuospatial function.

**Conclusion:**

Our study is one of few to examine the relationship between OUES, brain volume, and cognition. Though both OUES and peak VO_2peak_ were associated with visuospatial function, OUES was associated with more brain regions and brain tissue types. One possible interpretation is that OUES may account for more variance in CRF, making it less likely to be skewed by individual differences across participants and creating a more robust association with related brain and psychological testing measures. This could potentially make OUES a valuable measure for exercise interventions for improving brain health and cognition in clinical populations.

